# Analysis of the Incidence of Euthyroid Sick Syndrome in Comprehensive Intensive Care Units and Related Risk Factors

**DOI:** 10.3389/fendo.2021.656641

**Published:** 2021-06-09

**Authors:** Jianying Guo, Yanyan Hong, Zhiyong Wang, Yukun Li

**Affiliations:** ^1^ Department of Endocrinology, The Third Hospital of Hebei Medical University, Shijiazhuang, China; ^2^ Department of Critical Care Medicine, The Third Hospital of Hebei Medical University, Shijiazhuang, China; ^3^ Department of School Infirmary, Infirmary of Shijiazhuang Institute of Technology, Shijiazhuang, China

**Keywords:** low plasma triiodothyronine, nonthyroidal illness syndrome, thyroid-stimulating hormone, thyroid hormone, intensive care unit

## Abstract

**Objective:**

A low concentration of plasma triiodothyronine (T3) indicates euthyroid sick syndrome (ESS), which could be associated with a poor outcome in patients in intensive care units (ICUs). This study evaluated the relationship between ESS and prognostic indicators in patients admitted to an ICU and examined the free T3 (FT3) cut-off points that could be associated with 28-day mortality.

**Methods:**

This prospective observational study included patients admitted to the ICU of The Third Hospital of Hebei Medical University between February and November 2018. Baseline variables and data on the occurrence of low FT3 were collected. The patients were divided into ESS (FT3 < 3.28 pmol/L) and non-ESS groups. The relationship between ESS and prognostic indicators in patients admitted to the ICU was evaluated, and the FT3 cut-off points that could be associated with 28-day mortality were examined.

**Results:**

Out of a total of 305 patients, 118 (38.7%) were in the ESS group. Levels of FT3 (P < 0.001) and FT4 (P = 0.001) were lower, while the 28-day mortality rate (P < 0.001) and hospitalization expenses in the ICU (P = 0.001) were higher in the ESS group. A univariable analysis identified ESS, FT3, free thyroxine (FT4)/FT3, the APACHE II score, the sequential organ failure (SOFA) score, the duration of mechanical ventilation, creatinine (CREA) levels, the oxygenation index (HGB), white blood cells, albumin (ALB) levels, age, and brain natriuretic peptide (BNP) levels as factors associated with 28-day mortality (all P < 0.05). The cut-off value of FT3 for 28-day mortality was 2.88 pmol/L, and the 28-day mortality rate and hospitalization expenses in the ICU were higher in patients with ESS. The syndrome was confirmed to be independently associated with 28-day mortality.

**Conclusion:**

This study determined the incidence of ESS in the comprehensive ICU to be 38.7%. APACHE II, SOFA, BNP, APTT, HGB, PLT, CREA, ALB, FT4, SBP, and DBP are closely related to ESS, while BNP, PLT, and ALB are independent risk factors for the syndrome.

## Preface

Thyroid hormones act on various organ systems throughout the body and are essential for maintaining basic physiological activities. The concept of euthyroid sick syndrome (ESS) was proposed in the 1970s. It means that under the influence of many factors, including trauma, infection, surgery, and inflammation, patients with normal thyroid function are affected by a series of thyroid hormone level disorders. The main manifestations of ESS are a decrease in the level of triiodothyronine (T3) or free triiodothyronine (FT3), an increase in the level of thyroid hormone reverse T3, and normal or decreased levels of thyroxine (T4)/free T4 (FT4) and thyroid-stimulating hormone (TSH) (1). Because the decline in T3 is the most prominent and easy to detect in ESS, the disorder is also known as low T3 syndrome (2). Thyroid hormone level disorder is secondary to various clinical diseases because of the normal primary thyroid function. ESS is also known as nonthyroidal illness syndrome (NTIS) (2–8).

Intensive care units (ICUs) are dominated by critically ill patients, and ESS is a common disease in these facilities (2). Few studies have been conducted on the incidence of ESS and related factors in ICU patients, and the subject has received little attention. Studies are available on the incidence of ESS in ICUs, but most of the sample sizes are small, and the type of disease is relatively specific (9, 10). There has been a lack of investigation and in-depth analysis of large samples concerning the incidence of ESS in comprehensive ICUs. Therefore, this study aims to explore the incidence of ESS in a comprehensive ICU and to analyze and investigate the clinical risk factors of the syndrome.

## Materials and Methods

### Research Subjects

All eligible patients were admitted to the comprehensive ICU of the Third Hospital of Hebei Medical University between February 2018 and November 2018. This study was approved by the Ethics Committee of the Third Hospital of Hebei Medical University (approval No. 2018-019-1). After being informed of the study’s procedures, all patients or authorized family members signed a written informed consent form before enrolment.

### Inclusion Criteria

The inclusion criteria were as follows: (1) aged between 16 and 80 years, (2) admitted to the ICU for various reasons, and (3) normal past thyroid function with no history of thyroid disease, such as hyperthyroidism, hypothyroidism, or thyroid tumor. According to the level of thyroid hormone FT3, patients were divided into the ESS group and the non-ESS group. The normal value of FT3 is 3.28–6.47 pmol/L; patients with FT3 < 3.28 pmol/L were assigned to the ESS group, and those with FT3 ≥ 3.28 pmol/L were assigned to the non-ESS group.

### Exclusion Criteria

The exclusion criteria were as follows: (1) hospitalization in the ICU for less than 24 hours, (2) admission to the ICU for organ donation or organ function maintenance, (3) pregnant or lactating women, (4) presence of tumors with endocrine dysfunction, and (5) use of anti-thyroid drugs, T4, or long-term use of iodine-containing preparations, such as long-term oral amiodarone.

### Data Collection

The collection of all the clinical data in this study was carried out by full-time ICU doctors and respiratory therapists. The researchers uniformly trained the data collection personnel. The main data sources included on-site collection, case and nursing data, monitoring data, and related laboratory tests. The researcher performed/obtained an acute physiology and chronic health evaluation score (APACHE II), the Glasgow coma scale (GCS), and the sequential organ failure score (SOFA); these were reviewed by another researcher to ensure the accuracy of the scores.

The patient’s body temperature, respiration, heart rate, and blood pressure were measured upon admission to the ICU. The body temperature was measured with a mercury thermometer, and the remaining parameters were measured with a monitor (Philips MP series). When patients underwent invasive blood pressure monitoring, the invasive blood pressure was preferred as the blood pressure value. When invasive blood pressure monitoring was not performed, the patient’s non-invasive (cuff) blood pressure was used to record the systolic and diastolic blood pressures, respectively.

### Specimen Collection and Laboratory Testing

The blood samples required for the test were collected by a full-time ICU nurse *via* the peripheral or central vein. The blood sample required for arterial blood gas analysis was obtained either by percutaneous puncture through the radial or femoral artery or was drawn through the arterial catheter. When drawing through the arterial catheter, 5–10 ml of blood was drawn out in advance to prevent interference with the liquid in the pipeline. Arterial blood sampling was performed by a full-time ICU nurse. The enrolled patients were monitored for FT3, FT4, and TSH levels within 24 hours of admission to the ICU. The nuclear medicine physicians at the hospital used radioimmunoassays to analyze the blood samples (Beckman DXI800). The normal laboratory values of thyroid function in the hospital were: FT3: 3.28–6.47 pmol/L, FT4: 7.64–16.3 pmol/L, and TSH: 0.49–4.91 mIU/L.

### Scores

According to the standard requirements for each scoring scheme, the APACHE II, GCS, and SOFA score tests were performed on the enrolled patients; they were scored by a professional ICU doctor and reviewed by another physician. The scoring rules are shown in the attached table.

### Statistical Methods

All data were statistically analyzed using SPSS 24.0 software (IBM). Measurement data conforming to the normal distribution were expressed as $\bar{x}\pm s$, and measurement data not conforming to the normal distribution were expressed in the median form (the interquartile range). An independent sample t-test was used for the comparison of measurement data conforming to the normal distribution, and the Mann–Whitney rank sum test was used for the abnormal distribution. The count data were expressed as the number of cases (percentage), and Pearson’s Chi-squared test was used for comparison between the groups. Univariate and multivariate logistic regression were used to analyze the related risk factors, and the selection of multivariate logistic regression variables was based on the standard of P < 0.1. The significance level of all data adopted two-tailed approach in which a = 0.05.

## Results

### Comparison of Population Characteristics

A total of 305 eligible patients were included in the study, including 118 patients with ESS (defined as FT3 < 3.28 pmol/L), which accounted for 38.7% of the total patients, and 187 non-ESS patients, which accounted for 61.3% of the total. The 95% confidence interval for the incidence of ESS was 32.8%/44.6%. There was no significant difference in gender and age distribution between the ESS and the non-ESS groups (P > 0.05). The ESS group contained 82 males (69.5% of the total) and 36 females (30.5%), while the non-ESS group included 127 males (67.9% of the total) and 60 females (32.1%). The age of the ESS group was 57.5 ± 16.6 years, and the age of the non-ESS group was 54.0 ± 15.0 years.

### Comparison of Disease Type Composition Between the ESS and the Non-ESS Groups

The disease types included brain injury or diseases (71/305 cases, 23.3%), after operation (32/305 cases, 10.5%), severe disease resulting from medication (23/305 cases, 7.5%), sepsis or sepsis shock (31/305 cases, 10.2%), severe trauma (137/305 cases, 44.9%), tumor (9/305 cases, 3.0%), and pulmonary embolism (2/305 cases, 0.7%). The disease compositions of the ESS and non-ESS groups were different with a statistical difference of P < 0.001. In the ESS group, patients with severe trauma and sepsis accounted for relatively high proportions of the total at 37.3% and 21.2%, respectively, and patients with severe trauma and brain injury in the non-ESS group accounted for relatively high proportions of the total at 49.7% and 28.3%, respectively.

### Comparison of Scores Between the ESS and Non-ESS Groups

The APACHE II score of the ESS group was 15.44 ± 9.09, which was significantly higher than that of the non-ESS group (11.01 ± 6.78, P < 0.001). The SOFA score of the ESS group was 7 (0–19), which was significantly higher than in the non-ESS group [4 (0–21), P < 0.001]. Although the GCS score of the ESS group was 12.2 ± 4.5, which was higher than in the non-ESS group (12.2 ± 4.6), the difference was not statistically significant (P = 0.934) (see **Table 1**).

**Table 1:** Comparison of scores between ESS group and non-ESS group.

Score	ESS group	non-ESS group	*P -value*
APACHE II	15.44 ± 9.09	11.01 ± 6.78	<0.001
SOFA	7 (0-19)	4 (0-21)	<0.001
GCS	12.2 ± 4.5	12.2 ± 4.6	0.934

### Comparison of Vital Signs at Enrolment Between the ESS and Non-ESS Groups

At the time of enrolment, the body temperature of the ESS group was 37.33°C ± 0.92°C, while that of the non-ESS group was 37.26°C ± 0.84°C. The heart rate of the ESS group was 96.8 ± 34.1 beats per minute while in the non-ESS group, the figure was 92.7 ± 27.8 beats. The breathing rates of the ESS and non-ESS groups were 21.3 ± 8.2 and 22.8 ± 13.1 breaths per minute, respectively. There was no significant difference in body temperature, heart rate, or breathing between the two groups at the time of enrolment (P > 0.05). The systolic blood pressure of the ESS group was 118.6 ± 38.4 mmHg, while it was 127.9 ± 29.3 mmHg in the non-ESS group. The ESS group had a diastolic blood pressure of 64.8 ± 22.4 mmHg, while the non-ESS group’s figure was 70.2 ± 16.9 mmHg. Both the systolic blood pressure and diastolic blood pressure were lower in the ESS group than in the non-ESS group, and there was a statistical difference (P = 0.02).

### Comparison of Thyroid Hormone Levels Between the ESS and Non-ESS Groups

In this study, ESS was defined by the FT3 level. In the ESS group, the FT3 level was 2.72 ± 0.40 pmol/L, while the level in the non-ESS group was 3.97 ± 0.63 pmol/L. The FT3 level of the ESS group was significantly lower than that of the non-ESS group, and there was a statistical difference (P < 0.001). The FT4 level of the ESS group was 11.79 ± 2.83 pmol/L, while it was 13.59 ± 5.37 pmol/L in the non-ESS group. The FT4 level of the ESS group was lower than that of the non-ESS group, and there was a statistical difference (P = 0.001). The TSH levels of the ESS and non-ESS groups were 0.68 (0.41–1.54) and 0.69 (0.36–1.38) mIU/L, respectively, with no significant statistical difference (P = 0.148) (see **Table 2**).

**Table 2:** Comparison of thyroid hormone levels between ESS group and non-ESS group.

	ESS group	non-ESS group	*P -value*
FT3(pmol/L)	2.72 ± 0.40	3.97 ± 0.63	<0.001
FT4(pmol/L)	11.79 ± 2.83	13.59 ± 5.37	0.001
TSH(mIU/L)	0.68(0.41-1.54)	0.69(0.36-1.38)	0.573

### Comparison of Biochemical Indicators Between the ESS and Non-ESS Groups

The albumin (ALB) level of the ESS group was 26.63 ± 6.51 g/L, while it was 30.13 ± 7.13 g/L in the non-ESS group. The ALB level of the ESS group was significantly lower than that of the non-ESS group, and there was a statistical difference (P < 0.001). In the ESS group, the creatinine (CREA) level was 120.3 ± 165.8 umol/L, while the non-ESS group had a CREA level of 80.6 ± 85.8 umol/L. The ESS group’s CREA level was significantly higher than in the non-ESS group, and there was a statistical difference (P = 0.007). There was no significant difference in TBiL, blood Na +, and blood K + between both groups (P > 0.05).

### Comparison of Blood Routine Indices Between the ESS and Non-ESS Groups

The WBC level in the ESS group was 12.5 ± 7.56 × 109/L, while the WBC level was 11.6 ± 5.49 × 109/L in the non-ESS group. There was no statistical difference between the two groups (P = 0.253). The oxygenation levels of the ESS and non-ESS groups were 98.2 ± 25.15 and 108.0 ± 20.49 g/L, respectively, which was statistically different (P < 0.001). The PLT level of the ESS group was 128.9 ± 105.3 × 10^9^/L, while in the non-ESS group, it was 158.4 ± 97.2 × 10^9^/L, which represented a statistical difference (P = 0.013).

### Comparison of Blood Coagulation Indices Between the ESS and Non-ESS Groups

The PT level of the ESS group was 14.81 ± 11.83 s, and the level in the non-ESS group was 13.29 ± 4.56 s. There was no statistical difference between the two groups (P = 0.116). The APTT values in the ESS and non-ESS groups were 33.41 ± 16.65 and 29.97 ± 12.18 s, respectively. The APTT was longer in the ESS group than in the non-ESS group, and there was a statistical difference (P = 0.039). The FIB level in the ESS group was 3.35 ± 2.61 g/L, while it was 3.32 ± 1.48 g/L in the non-ESS group. There was no statistical difference between the groups (P = 0.907).

### Comparison of Brain Natriuretic Peptide (BNP) and PCT Indicators Between the ESS and Non-ESS Groups

The BNP levels in the ESS group were 212.5(84.5-1200.0)pg/mL, while the BNP levels in the non-ESS group were 76.0(18.0-259.0)pg/mL; the levels in the ESS group were significantly higher than in the non-ESS group, which was a statistical difference (P = 0.002). The PCT levels in the ESS and non-ESS groups were 1.02(0.28-2.99)ug/L and 0.32(0.06-1.49)ug/L, respectively. The PCT level in the ESS group was higher than in the non-ESS group, and there was no statistical difference (P = 0.056).

### Comparison of Blood Gas Analysis Indices Between the ESS and Non-ESS Groups

The Lac level of the ESS group was 1.50(1.00-3.10) mmol/L, while it was 1.40(0.90-2.60) mmol/L in the non-ESS group, and there was no statistical difference (P = 0.101). The PH levels of the ESS and non-ESS groups were 7.20 ± 1.17 and 7.39 ± 0.64, respectively. The ESS group had a lower PH level than the non-ESS group, and there was no statistical difference (P = 0.075). The BE level of the ESS group was –2.72 ± 6.36 mmol/L, while it was –1.57 ± 4.68 mmol/L in the non-ESS group. There was no statistical difference (P = 0.08). The PCO2 levels in the ESS and non-ESS groups were 36.7 ± 14.4 and 34.4 ± 9.7 mmHg, respectively, with no statistical difference (P = 0.146). The oxygenation index (HGB) level of the ESS group was 273.0 ± 145.7, while that of the non-ESS group was 281.1 ± 136.2. There was no statistical difference (P = 0.631).

### Logistic Regression Analysis of ESS-Influencing Factors

A univariate logistic regression analysis was performed on the variables between the ESS and the non-ESS groups. It was found that APACHE II, SOFA, BNP, APTT, HGB, PLT, CREA, ALB, FT4, SBP, and DBP are closely associated with ESS, and all have a P value of < 0.05. The odds ratio (OR) values of APACHE II, SOFA, BNP, APTT, and CREA are all greater than 1 at 1.074, 1.143, 1.001, 1.017, and 1.003, respectively. OR > 1 indicates that the greater the index value, the higher the risk of ESS. The OR values of HGB, PLT, ALB, FT4, SBP, and DBP are 0.98, 0.997, 0.929, 0.864, 0.992, and 0.985, respectively. OR < 1 indicates that the lower the index value, the higher the risk of ESS.

A multivariate logistic regression analysis was performed on the above covariates selected by univariate regression analysis. Among them, the scores for APACHE II and SOFA are recognized as the prognostic scoring systems of the disease. The content includes HGB, CREA, blood pressure, and other variables, and it is no longer included in the multivariate regression analysis. The entry method was used for analysis, with P > 0.1 as the exclusion criterion, and the covariates BNP, APTT, HGB, PLT, CREA, ALB, FT4, SBP, and DBP were included. The results show that BNP, PLT, and ALB are independent risk factors for the occurrence of ESS (P ≤ 0.05); their OR values are 1.001, 0.994, and 0.931, respectively (see **Table 3**).

**Table 3:** Multivariate Logistic regression analysis of ESS influencing factors.

Variable	β	SE	Wald	P	OR	95%CI	
BNP	0.001	0	3.834	0.05	1.001	1	1.001
APTT	0.005	0.016	0.104	0.747	1.005	0.974	1.038
HGB	-0.003	0.009	0.132	0.716	0.997	0.98	1.014
PLT	-0.006	0.003	4.702	0.03	0.994	0.988	0.999
CREA	-0.001	0.002	0.136	0.712	0.999	0.995	1.003
ALB	-0.072	0.036	4.009	0.045	0.931	0.867	0.998
FT4	-0.078	0.064	1.481	0.224	0.925	0.816	1.049
SBP	0.001	0.011	0.014	0.907	1.001	0.979	1.024
DBP	-0.019	0.021	0.793	0.373	0.981	0.942	1.023

## Discussion

This study evaluated the relationship between ESS and prognostic indicators in patients admitted to an ICU and found the following results. First, ESS was independently associated with 28-day mortality, and the cut-off value of FT3 for 28-day mortality was 2.88 pmol/L. Second, FT4, APACHE II, SOFA, SBP, DBP, BNP, APTT, HGB, PLT, CREA, and ALB are closely linked to ESS. Finally, BNP, PLT, and ALB are independent risk factors for ESS.

In 2018, a study of thyroid hormones and prognoses involving 360 patients with sepsis was published in India. In this study, the lower limit of the normal value of FT3 was 3.7 pmol/L; values below this were defined as ESS (11). In another study related to thyroid hormone and mortality involving 270 cases of internal medicine ICU patients, the lower limit of the FT3 normal value was 3.5 pmol/L (12). In Gary et al.’s (13) 2019 study of the relationship between ICU patients with invasive mechanical ventilation and FT3, the lower limit of the normal FT3 value was 2.3 pg/mL, and FT3 < 2.3 pg/mL was defined as the ESS group. In this study, the lower limit of the serological normal value of FT3 was 3.28 pmol/L, which is the same value found in the standard definition of ESS.

The results of this study show that the incidence of ESS in comprehensive ICUs is 38.7%. In 2007, a retrospective study of 247 patients in an internal medicine ICU in Germany (14) found that 97 patients had a decline in FT3 levels; ESS accounted for 44.1% of patients, of which 20.5% also had a decline in their levels of FT4. In this study, patients with severe trauma accounted for a relatively high proportion of total patients, the APACHE II score level was relatively low, and the incidence of ESS was 38.7%, which is slightly lower than in the German study. In 2012, Critical Care published a study on FT3 and the prognostic values of 480 ICU patients in China. The results showed a decline in FT3 in 261 cases; 23 of these had a decrease in total T3, 53 cases had a drop in total T4, and 48 cases had a decline in FT4 levels. Among all thyroid hormone indicators, FT3 is the most sensitive indicator for ESS, and taking FT3 as the standard, the incidence of ESS in their study was 54.38% (6), which was higher than 38.7% in our study. Their study (6) focused on cardiovascular disease in elderly people with a mortality rate of 19.7%, which was also significantly higher than this study. This study better represents the general situation regarding integrated ICUs, and the results better reflect the incidence of ESS in integrated ICUs. However, this study only observed changes in thyroid hormone levels and the incidence of ESS at the time of enrolment. Therefore, the dynamic variations in thyroid hormones during ESS changes should be explored further.

The mechanism of ESS is still not fully understood. Various diseases cause abnormalities in the production, transport, and metabolism of thyroid hormones, which can lead to ESS; these include deiodinase activity, TSH secretion, binding of thyroid hormone to thyroid binding protein, transport to peripheral tissues, intranuclear receptor activity, and TSH secretion (1). Often, ESS is accompanied by changes in other endocrine systems, such as decreased serum gonadotropin/sex hormone concentrations and increased serum ACTH/glucocorticoid levels (15).

ESS is related to changes in the systemic immune and endocrine systems. The current literature reports that the specific mechanisms of ESS mainly include changes in thyroid hormone metabolism (16), TSH secretion (17), serum thyroid hormone binding protein (18), transmembrane transport of thyroid hormone (19), and changes in thyroid hormone nuclear receptors (20). Therefore, in ESS, various factors lead to abnormal body active regulation and abnormalities in thyroid hormone metabolism, regulation, transportation, transmembrane transport, and receptor binding. ESS is characterized by a change in thyroid hormones; however, its specific mechanism requires further study.

Much of the literature shows that ESS is significantly correlated with the severity of the disease, and the decline in FT3 levels is used to evaluate mortality and other prognostic markers (21, 22). In the current study, the APACHE II and SOFA scores in the ESS group are significantly higher than in the non-ESS group. The ESS group has a significant correlation between the severity of disease APACHE II score and the SOFA score. The univariate regression analysis results produced OR values for the APACHE II and SOFA scores of 1.047 and 1.143, respectively, in terms of the occurrence of ESS. This indicates that as the score increases, the risk of ESS also rises. In addition, the age of the ESS group in this study is higher than that of the non-ESS group. The univariate regression analysis shows no correlation between ESS and age; therefore, the correlation between these factors requires further investigation (23–25).

The current study included the basic vital signs of body temperature, heart rate, respiration, and blood pressure upon admission to the ICU. The results show that there is no significant difference in body temperature, heart rate, or respiration between the ESS and non-ESS groups. ESS is closely correlated with blood pressure. The diastolic and systolic blood pressures of the ESS group are significantly lower than those of the non-ESS group. A drop in blood pressure often indicates shock, insufficient tissue perfusion, etc., causing severe metabolic disorders and systemic inflammatory reactions (26). Often, a drop in blood pressure during shock indicates that the shock has entered a decompensated period. At this stage, the inflammatory response is severe, which seriously affects the metabolism of endocrine hormones, such as thyroid hormones (27). Therefore, blood pressure is one of the risk factors related to ESS.

This study explored the correlation between common physiological and biochemical indicators and ESS. A statistical analysis confirmed that BNP, APTT, HGB, PLT, CREA, and ALB are closely related to ESS. Meanwhile, a univariate logistic regression analysis in this study confirmed that the risk of ESS is positively associated with BNP, APTT, and CREA (OR >1), and it is inversely related to HGB, PLT, and ALB (OR < 1). A multivariate regression analysis revealed that BNP, PLT, and ALB are independent risk factors for ESS. ALB is known to be the main protein in plasma. T3 levels decline in the early stage of ESS, and the decrease of T4 to T3 conversion is related to the reduction in ALB levels (1).

In severe trauma, PLT is an important indicator of blood loss. Studies have reported significant decreases in the levels of T4 and FT4 in patients with massive blood loss following trauma (28). BNP is a heart failure marker; elevated BNP levels reflect heart involvement, and ESS is closely related to heart failure. A 2020 study included 956 patients with acute heart failure, and ESS occurred in 511 cases (29, 30). Therefore, BNP, PLT, and ALB may become important indicators for the early detection and identification of ESS. However, as far as the authors are aware, there are no similar studies or reports on the aforementioned risk factors for ESS in the literature, so the results of the current study require further investigation.

This study has certain limitations. First, the number of research samples is relatively small. The distribution of disease types is quite diverse, and different types of patients were analyzed together. For further research on the incidence of ESS in ICUs, it is necessary to expand the number of samples and balance the types of diseases. Second, the data collected in this research were obtained upon admission to the ICU, and no dynamic monitoring was performed. Finally, the variables included in this study’s multivariate regression analysis might not be comprehensive, and further research is needed.

## Conclusion

This study found the ESS incidence rate in the comprehensive ICU to be 38.7%. APACHE II, SOFA, BNP, APTT, HGB, PLT, CREA, ALB, FT4, SBP, and DBP are all closely related to ESS, and BNP, PLT, and ALB are independent risk factors for the syndrome.

## Data Availability Statement

The original contributions presented in the study are included in the article/supplementary material. Further inquiries can be directed to the corresponding authors.

## Author Contributions

Conception and design of the research: JG and YL. Acquisition of data: YH and ZW. Analysis and interpretation of the data: JG and YH. Statistical analysis: JG and ZW. Obtaining financing: None. Writing of the manuscript: JG and YL. Critical revision of the manuscript for intellectual content: JG and YL. All authors contributed to the article and approved the submitted version.

## Funding 

Key scientific and technological research plan of Hebei Province (20190626).

## Conflict of Interest

The authors declare that the research was conducted in the absence of any commercial or financial relationships that could be construed as a potential conflict of interest.
